# Microbiota Influences Morphology and Reproduction of the Brown Alga *Ectocarpus* sp.

**DOI:** 10.3389/fmicb.2016.00197

**Published:** 2016-02-24

**Authors:** Javier E. Tapia, Bernardo González, Sophie Goulitquer, Philippe Potin, Juan A. Correa

**Affiliations:** ^1^CNRS, Université Pierre-et-Marie-Curie, UMI 3614, Biology and Ecology of Algae, Station Biologique de RoscoffRoscoff, France; ^2^Departamento de Ecología, Facultad de Ciencias Biológicas, Pontificia Universidad Católica de ChileSantiago, Chile; ^3^Facultad de Ingeniería y Ciencias, Universidad Adolfo Ibáñez – Center of Applied Ecology and SustainabilitySantiago, Chile; ^4^MetaboMer Mass Spectrometry Core Facility, Université Pierre-et-Marie-Curie, CNRS, FR2424, Station Biologique de RoscoffRoscoff, France; ^5^Université Pierre-et-Marie-Curie, CNRS UMR 8227, Integrative Biology of Marine Models, Station Biologique de RoscoffRoscoff, France

**Keywords:** microbiota, bacteria–algae interaction, *Ectocarpus*, bacterial isolate, algal morphology, exometabolome

## Abstract

Associated microbiota play crucial roles in health and disease of higher organisms. For macroalgae, some associated bacteria exert beneficial effects on nutrition, morphogenesis and growth. However, current knowledge on macroalgae–microbiota interactions is mostly based on studies on green and red seaweeds. In this study, we report that when cultured under axenic conditions, the filamentous brown algal model *Ectocarpus* sp. loses its branched morphology and grows with a small ball-like appearance. Nine strains of periphytic bacteria isolated from *Ectocarpus* sp. unialgal cultures were identified by 16S rRNA sequencing, and assessed for their effect on morphology, reproduction and the metabolites secreted by axenic *Ectocarpus* sp. Six of these isolates restored morphology and reproduction features of axenic *Ectocarpus* sp. Bacteria-algae co-culture supernatants, but not the supernatant of the corresponding bacterium growing alone, also recovered morphology and reproduction of the alga. Furthermore, colonization of axenic *Ectocarpus* sp. with a single bacterial isolate impacted significantly the metabolites released by the alga. These results show that the branched typical morphology and the individuals produced by *Ectocarpus* sp. are strongly dependent on the presence of bacteria, while the bacterial effect on the algal exometabolome profile reflects the impact of bacteria on the whole physiology of this alga.

## Introduction

Plants and animals are associated with their microbiota, a complex assortment of microorganisms. As example, in the human gut, bacteria play a major role in stimulating immune system development (Lee and Mazmanian, 2010; Littman and Pamer, 2011). Recently, the communication between gut microbiota and the central nervous system has been established (Mayer, 2011), along with the emerging concept of a microbiota-gut-brain axis (Cryan and Dinan, 2012). Similarly, plant roots are colonized by a large diversity of soil microorganisms which are capable of producing beneficial (although sometimes negative, pathogenic, and presumably mostly neutral) effects on the plant (Newton et al., 2010). Plant growth-promoting bacteria (PGPB) stimulate growth by increasing photosynthetic capacity (Zhang et al., 2008), increasing tolerance to abiotic stress (Yang et al., 2009), by suppressing plant diseases (Choudhary and Johri, 2009; Van der Ent et al., 2009) and herbivory by insects (Van Oosten et al., 2008), among several other relatively poorly understood mechanisms/functions/processes.

On comparative grounds, plant and animal-bacteria interactions have received more attention than other macroorganisms-microorganisms interactions. In aquatic environments, microorganisms are quite abundant. It is estimated that, on average, one milliliter of seawater contains more than 10^6^ bacteria (Harder, 2009). In addition, marine environments favor formation of biofilms on diverse surfaces, including those of macroalgae (Weinberger, 2007), and other marine macroorganisms (Qian et al., 2007).

In this context, it is known that seaweeds interact with marine microorganisms throughout their life cycle (Goecke et al., 2012). The microbial communities inhabiting macroalgae are highly complex, dynamic and are constituted by a variety of microorganisms where bacteria are better described in terms of their diversity and function (Corre and Prieur, 1990; Burke et al., 2011a,b). In this interaction, macroalgae represent an excellent environment for bacterial colonization and reproduction by providing nutrients and a suitable surface for attachment (Armstrong et al., 2000; Singh and Reddy, 2014). The advantages for the algal host have been also described during recent years. Bacteria can mineralize organic substrates giving the algae carbon dioxide, minerals and growth factors (Matsuo et al., 2005). Other studies have shown that marine bacteria produce nitrogen compounds that are a source of nutrients for algae. For example, the nitrogen supply of *Caulerpa taxifolia* is provided by an endophytic bacteria from the *Agrobacterium-Rhizobium* group, which lives in the rhizoids of this algae (Chisholm et al., 1996).

In addition to the nutritional benefits, it has been shown that the presence of certain bacteria is needed for normal morphological development and growth of some green (Matsuo et al., 2003; Marshall et al., 2006; Spoerner et al., 2012) and red macroalgae (Singh et al., 2011; Fukui et al., 2014). Moreover, associated bacteria are known to induce settlement of zoospores of *Ulva* species and release of spores from *Acrochaetium* sp. (Joint et al., 2007; Weinberger et al., 2007).

The above information has been obtained mainly based on studies using green and red algal species, leaving aside the important group of brown algae. The *Phaeophycean* taxa is one of the more diverse groups of macroalgae (Andersen, 2004) and possesses significant ecological roles in coastal ecosystems (Cock et al., 2011).

Brown algae are phylogenetically distant not only from terrestrial plants, animals and fungi, but also from red and green algae (Baldauf, 2003). Indeed, they differ in many aspects of their biology with respect to the other algal groups. Some of these differences correspond to: composition and pathways of cell wall synthesis (Nyvall et al., 2003), their ability to synthesize C18 and C20 oxylipins (Ritter et al., 2008), in their ability to accumulate iodine (Kupper et al., 2008), among several others. Bacteria have been described living in association with brown algae (Hengst et al., 2010; Lachnit et al., 2011), and there are some early observations linking bacterial presence with normal development and growth of these organisms (Pedersen, 1968).

In order to elucidate basic aspects of the biology of brown algae, a small species with a filamentous structure, *Ectocarpus siliculosus*, has been chosen as a model (Peters et al., 2004). Several molecular tools and databases are now available for this algae including its complete genome sequence (Cock et al., 2010), genetic maps (Heesch et al., 2010), transcriptomics (Le Bail et al., 2008a; Dittami et al., 2009) and proteomics (Contreras et al., 2008) approaches. Despite a diverse array of studies addressing its life cycle (Coelho et al., 2011a,b; Arun et al., 2013), acclimation to biotic and abiotic stress (Dittami et al., 2011; Grenville-Briggs et al., 2011), morphological development (Le Bail et al., 2010, 2011) and genetic diversity on the field (Peters et al., 2010), to date there is limited knowledge on the interactions between *Ectocarpus* and its associated microbiota. Recently, Dittami et al. (2015) described how *Ectocarpus* associated bacteria are essential for acclimation to salinity gradients, showing the importance of these microorganisms to the alga under stress conditions. More than 40 years ago, Pedersen (1968) also reported a potential role for bacteria in the development of members of this algal genus. She described that axenic cultures of *E. fasciculatus* showed slow growth and atypical development when kept under sterile, axenic conditions, suggesting an influence of bacteria for the normal growth and development of these algae.

A more detailed evaluation of the role of bacteria on brown algae development and physiology is clearly required in order to establish and understand the influence of these microorganisms and the mechanisms involved in this interaction. The present study describes the isolation of bacteria and the evaluation of their role as regulators of morphology and reproduction of the brown algal model *Ectocarpus* sp. [strain Ec32 formerly referred as *E. siliculosus* (Peters et al., 2010)]. The effects of bacterial inoculation and bacterial exudates were determined, and proved to be essential in shaping the development and reproduction of this algal model. The impact of bacterial presence on the metabolites secreted by the alga, as an approach to understand the bacterial influence on the general metabolism of the host (Macel et al., 2010; Goulitquer et al., 2012), was also assessed. The result of this approach revealed that colonization of axenic *Ectocarpus* sp. with single bacterial species drives a major impact in the algal exometabolome profile, highlighting the effect of bacteria on the whole physiology of this alga.

## Materials and Methods

### Culture of Axenic *Ectocarpus* sp.

The experiments were carried out using the axenic laboratory cultures of haploid *Ectocarpus* sp. parthenosporophyte isolate Ec 32 (Culture Collection of Algae and Protozoa accession no. 1310/4; origin, San Juan de Marcona, Perú), which was produced by germination of unfertilized gametes (Le Bail et al., 2008b). Axenization of algal individuals was carried out according to Müller et al. (2008). Briefly, small *Ectocarpus* fragments were placed around antibiotic disks on Zobell medium. Four weeks later, algal fragments from bacteria-free areas were taken and put into Petri dishes with sterilized natural seawater. After another 4 weeks, some of the fragments were put on Zobell medium to check for bacterial growth while others were checked for bacterial presence by microscopy. Fragments from bacterial-free algal material were then transferred to Petri dishes with SFC culture medium (Correa and McLachlan, 1991) for growth and experimentation. Individuals were grown in 12-mL Petri dishes in sterile-pasteurized SFC medium in a controlled-environment cabinet at 13°C with a 12:12-h light:dark cycle (light intensity of 30 μmol photons m^-2^ s^-1^). All growth treatments were performed according to these conditions.

### Axenicity Controls

**FIGURE 1:**
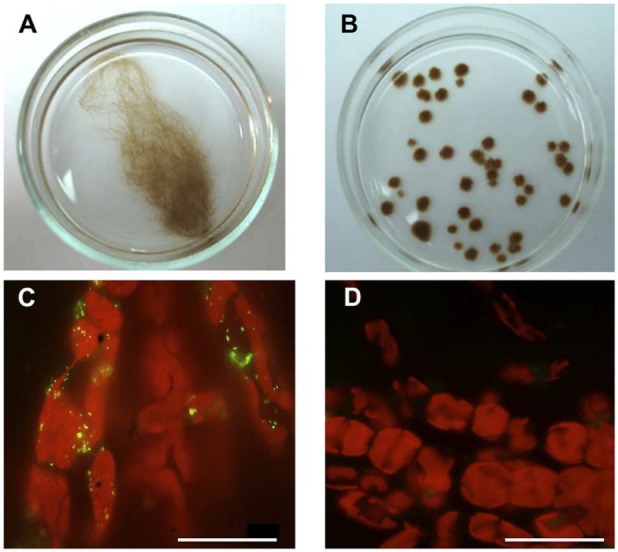
**Morphological differences between *Ectocarpus* sp. individuals growing under axenic and unialgal culture conditions.** **(A,B)** Examples of 2 month-old *Ectocarpus* sp. individuals growing in sterilized SFC medium. **(A)** *Ectocarpus* sp. individual showing its characteristic regular branched morphology in a unialgal culture. **(B)** Axenic *Ectocarpus* sp. individuals showing the atypical “small ball-like” appearance. **(C,D)** Detection of bacteria on *Ectocarpus* sp. surface (red, chloroplasts autofluorescence), by epifluorescence microscopy visualization using green-yellow, SYBR green II staining. **(C)** Bacteria detected on the filament surface of an *Ectocarpus* sp. individual grown in a unialgal culture. **(D)** Absence of bacteria on the surface of an axenic *Ectocarpus* sp. individual. All bars, 30 μm.

In order to check for axenicity and cross-contamination, the following approaches were used:

(1) Visualization of bacteria on *Ectocarpus* surface at the beginning and the end of each treatment. Algal individuals were washed twice with sterile seawater and then exposed for 10 min to sterile seawater containing 0.22 μm filter-sterilized SYBR Green II. Observations were performed with an Olympus BX60 (Tokyo, Japan) epifluorescence microscope. See results of this approach (**Figures 1C,D** and **4D** and **Supplementary Figure S1**).

(2) DNA extraction from the treatment supernatants, PCR amplification of the 16S rRNA gene and AluIII restriction of the amplicons obtained. With this method it was possible to check bacterial presence (positive amplification) and also if the bacterial treatments were contaminated with other bacteria, by looking at the digestion profiles of the amplicons (see results of this approach in **Supplementary Figure S1**). 16S rRNA gen amplification and amplicon digestion procedures were also performed with DNAs from the bacterial isolates in order to compare the digestion profiles with those obtained from supernatants at the beginning and at the end of each treatment.

(3) To add supernatant of the treatments or *Ectocarpus* individuals that were exposed to bacterial isolates, to bacterial culture media (Zobell broth) and observe the growth of microorganisms after 2 weeks. See results of this approach in (**Supplementary Figure S2**).

### Seawater Microorganisms Effect on Axenic *Ectocarpus* sp.

In order to evaluate the influence of microorganisms on *Ectocarpus* sp. SFC medium using natural seawater (SW) coming from two different places: Caleta Maitencillo (32° 39′ S, 71° 29′ W) and Las Cruces (33° 30′ S, 71° 37′ W) were prepared. Seawater from Las Cruces was obtained in two different seasons, summer and winter. The SW was filtered using a 3 μm pore size filter (Merck Millipore, Darmstadt, Germany) so bacteria, some unicellular fungi and fungal spores were still present. To check for the presence of bacteria, 10 μL of filtered SW were plated on Zobell agar and after 3 days of incubation at 20°C microbial growth was clearly observed. To assess the effect of microbes containing SFC medium, four axenic *Ectocarpus* sp. individuals per plate (three plates) were exposed to 12 mL of this medium. Each experiment was performed in triplicate using these three different media. The spores produced that were settled and germinated after 7 days, were counted (30 random observations in a 1 mm^2^ area each). After 3 weeks, the percentage of individuals with upright filaments was determined (50 individuals per replicate). Observations were made in a Nikon Optiphot-2 microscope. To test the presence of *Ectocarpus* spores, running controls of filtered SW culture medium without the alga were performed along with each experiment. No *Ectocarpus* individuals were detected in any of these controls.

### Isolation of Bacteria from *Ectocarpus* Individuals Maintained in Unialgal Cultures

Bacteria were isolated from the surfaces of *Ectocarpus* unialgal strains Ec 32 (mentioned above) and Ec 524 (Culture Collection of Algae and Protozoa accession 1310/333, origin Caleta Palito, Chile 26°15′S, 70°14′W). Both strains were maintained under laboratory conditions as described above and always displayed a filamentous morphology. To isolate bacteria, small algal pieces were gently washed twice in sterile seawater, then grinded and spread on three different marine agar media: marine broth (Zobell) supplemented with 1.5% agar; sterile natural seawater, obtained by filtration and pasteurization, supplemented with 1.5% agar; and seawater R2A agar (Suzuki et al., 1997). The dishes were incubated at 20°C for 10 days and individual colonies were picked off and streaked onto the agar from which they were isolated in order to obtain single colonies. Bacterial isolates were maintained at 4°C while they were used, stocks were passed to -70°C in glycerol to conserve them.

### Identification of Bacterial Isolates by 16S rRNA Genes Sequencing

DNA from bacterial isolates was obtained using the PureLink^®^ Genomic DNA Mini Kit (Life Technologies, Carlsbad, CA, USA) according to the manufacturer’s instructions. PCR amplification of partial 16S rRNA gene sequences were carried out using the forward primer 8f (5′-AGATTTGATCCTGGCTCAG-3′) and the reverse primer 1492r (5′-GGTTACCTTGTTACGACTT-3′) (Weisburg et al., 1991). Sequencing was carried out at Macrogen Inc. (Seoul, Korea). A search for 16S rRNA similarities of sequences from isolated bacteria was made with the BLAST tool available online^1^. 16S rRNA gene sequences of bacterial isolates have been deposited at GenBank under accession numbers provided in **Supplementary Table S1**.

### Screening the Effects of Bacteria on Axenic *Ectocarpus* sp. Morphology and Reproduction

Axenic *Ectocarpus* sp. individuals were exposed to 12 mL of pasteurized SFC medium (four individuals per plate). Pasteurized medium, 95°C for 30 min followed by 90 min at 72°C, was preferred over autoclaved medium to avoid some salt precipitation during the sterilization process. Bacterial isolates were used in a density of approximately 10^7^ cells per milliliter in pasteurized SFC medium. Control (individuals without bacteria) and treatment plates were incubated according to the conditions mentioned above. Growth medium was replaced by sterile fresh material every 7 days. To determine the effect on morphology, individuals grown for 21 days after germination were evaluated according to the presence or absence of upright filaments (50 individuals per analysis, the analysis was repeated three times). To evaluate reproduction, the number of individuals produced 6 weeks after germination was counted in 30 random observations in a 1 mm^2^ area each (10 observations per plate). Observations were made in a Nikon Optiphot-2 microscope. Experiments were performed in three replicates for each of the nine isolates tested.

When evaluating the presence or absence of upright filaments, 20 random individuals were chosen to analyze filament and elongated cells sizes. Three cells per individual were evaluated. Cell sizes were measured with the ImageJ software^2^.

### Effect of Bacterial Growth Culture Supernatants and Bacteria-*Ectocarpus* Co-culture Supernatants on Axenic *Ectocarpus* Morphology and Reproduction

To obtain bacterial growth culture supernatants, each bacterial isolate was cultivated in 50 mL of sterile SFC medium in a 500 mL flask supplemented with 1% (w/v) glucose until they reached a density of approximately 10^7^ cells per milliliter in a shaker at 15°C. Bacterial cultures were centrifuged (30 min, 5000 × *g*) and the supernatant was filtered twice through 0.22 μm pore size filters (Merck Millipore, Darmstadt, Germany). The supernatants were used immediately. The experimental cultures media were refreshed every week using fresh bacterial supernatant.

To obtain bacteria-*Ectocarpus* co-culture supernatants, 1-week old media from direct bacterium inoculation treatments were used. Media from bacteria-*Ectocarpus* co-cultures were centrifuged and filtered the same way as bacterial supernatants. The obtained co-culture supernatants (approximately 12 mL) were directly exposed to axenic *Ectocarpus*, as previously mentioned. The effect on morphology and reproduction was evaluated as indicated in the corresponding section above. The experimental cultures were refreshed every week using 1-week old co-cultures supernatants. Supernatants from 1-week old axenic *Ectocarpus* cultures along with bacterial and algal culture media were used as controls. In order to check that bacterial supernatants and 1-week old co-cultures supernatants were not depleted of essential nutrients to sustain *Ectocarpus* growth, we placed individuals from unialgal and axenic cultures under these conditions and we compared them with their growth under starvation stressing conditions: natural seawater (NSW) without addition of any supplementary nutrient. While individuals from unialgal cultures developed normally, axenic *Ectocarpus* had an arrested growth and did not develop upright filaments and did not produce any new individuals (**Supplementary Figure S3**).

### Analysis of the Exometabolome

Exudate extracts were obtained by Solid Phase Extraction. Triplicates of 200 mL culture medium from axenic *Ectocarpus* sp. and bacterial isolate Z3 growing together plus exudate from both but growing alone were slowly passed through C18 cartridges (Sep Pak 6 mL, 1 g, Waters, Saint-Quentin en Yvelines, France) using an automated Dionex AUTO Trace 280 instrument (Thermo Fisher Scientific, Bremen, Germany). After washing with 5 mL of deionized water, the Sep Pak cartridges were dried under a nitrogen flux and then eluted in glass vials with 4 mL dichloromethane, followed with 4 mL methanol.

Ultra-high pressure liquid chromatography analysis of these extracts was performed using an RSLC Ultimate 3000 from Dionex (Thermo Fisher Scientific, Bremen, Germany) equipped with a quaternary pump and autosampler. Separations were achieved using an Acclaim RSLC 120 C18 1.9 μm (2.1 mm × 100 mm) column (Dionex) operated at 20°C, using 5 μL injection volume and a flow-rate of 250 μl min^-1^. Mobile phase A was composed of 0.1% acetic acid in MiliQ H_2_O, and mobile phase B was 0.1% acetic acid in acetonitrile. The gradient consisted of an initial hold at 20% mobile phase B for 2 min, followed by a linear gradient to 100% B in 8 min and a hold for 14 min, followed by re-equilibration for 6 min at 20% B, in a total run time of 30 min.

Mass spectrometry was performed using a LTQ-Orbitrap Discovery^TM^ mass spectrometer (Thermo Fisher Scientific, Bremen, Germany). Scans were collected in both positive and negative ESI mode over a range of *m/z* 50–1000. Ionization parameters were set as follows: sheath gas 5 psi, auxiliary gas 5 (arbitrary units), sweep gas 0 (arbitrary units), spray voltage 2.7 kV, capillary temperature 300°C, capillary voltage 60 V, tube lens voltage 127 V and heater temperature 300°C. The Xcalibur 2.1 software (Thermo Fisher Scientific) was used for instrument control and data acquisition. Following their acquisition, metabolomic fingerprints were deconvoluted to allow the conversion of the three-dimensional raw data (m/z, retention time, ion current) to time- and mass-aligned chromatographic peaks with associated peak areas. Massmatrix File Conversion tools were used to transform the original Xcalibur data files (^∗^.raw) to a more exchangeable format (^∗^.mzXML). Raw files were converted to the mzXML format using MassMatrix File Conversion Tools (Version 3.9, April 2011). Data were processed by the open-source XCMS software (Smith et al., 2006) running under R or on the online version, and further annotated by CAMERA^3^.

### Statistical Analysis

Data for number of spores produced and percentage of germinated individuals in sterile and non-sterile tests were compared using a two-sample *t*-test run on Minitab software version 16.1. Asterisk (^∗^) indicates differences on at least 5% level of significance (*p* < 0.05). Data for number of spores produced in alga-bacteria co-cultures were compared using a two-sample *t*-test run on Minitab software version 16.1. Different letters were used to indicate means that differ significantly (*p* < 0.05). For experiments addressing the effect of bacterial growth culture supernatants and bacteria-*Ectocarpus* co-culture supernatants on axenic *Ectocarpus* morphology and reproduction, univariate and multivariate analyses with a Tukey’s *post hoc* test, run on Minitab software version 16.1, were used for testing differences in individuals produced between treatments. Different letters were used to indicate means that differ significantly (*p* < 0.05). Multivariate statistical analyses of metabolite data were carried out using SIMCA-P (12.0.1, Umetrics, Umeå, Sweden). Data were log10-transformed and normalized using Pareto scaling. Principal Component Analysis (PCA) was carried to compare the intensity of mass/retention time pairs between the chromatograms.

## Results

### Differences in *Ectocarpus* sp. Morphology Growing in Unialgal or Axenic Culture Conditions

*Ectocarpus* sp. strain Ec32 in unialgal culture conditions displays its typical branched morphology (**Figure 1A**). When cultured under axenic conditions, *Ectocarpus* sp. shows a small ball-like appearance (**Figure 1B**), which differs from its branched natural morphology. The only difference between these two culture conditions is that the individuals in unialgal culture conditions still possess normally associated bacteria (**Figure 1C**) while under axenic conditions, individuals were previously treated with antibiotics to remove the associated microbiota (**Figure 1D**). Thus, bacterial absence in *Ectocarpus* cultures produces abnormal algal development.

### Effect of Seawater Microorganisms on Morphology and Reproduction of *Ectocarpus* sp.

**FIGURE 2:**
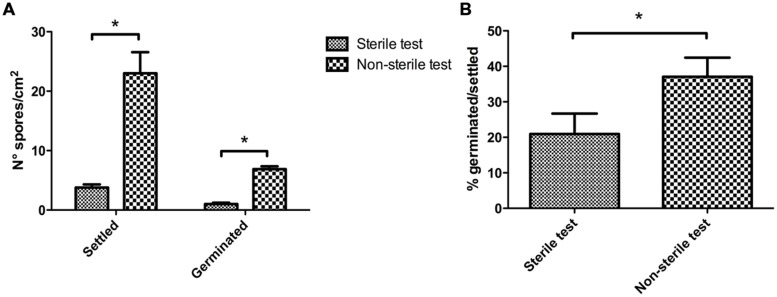
**Effect of seawater microorganisms on axenic *Ectocarpus* sp. spore production and germination.** **(A)** Number of spores settled and germinated after 7 days of axenic *Ectocarpus* sp. cultivation in sterile SFC (sterile test), and non-sterile SFC (non-sterile test) media. **(B)** Percentage of spore germinated related to the spores settled observed in **(A).** These determinations were repeated three times with similar results. The ^∗^ indicates means statistically different at *p* < 0.05.

In order to test whether microorganisms affect morphology and reproduction of *Ectocarpus* sp. a first approach was to expose axenic individuals to the presence or absence of microorganisms. Culture media prepared with surface seawater samples taken from two different coastal places were evaluated. First, these seawater samples were filtered, first, using a 3 μm pore size filter to prepare culture media still containing microorganisms, to perform the non-sterile tests. Then, the same seawater samples were filtered again using a 0.22 μm pore size filter and pasteurized in order to obtain the appropriate culture media to perform the sterile tests. After 1 week, axenic individuals proliferating in culture media containing microorganisms produced more algal spores than those individuals grown on sterile culture media (**Figure 2A**). The settled spores average counted in non-sterile tests was 23 per cm^2^ in contrast to an average of four settled spores per cm^2^ found under sterile conditions. Considering the spores already germinated (more than two cells), differences were also significant as in non-sterile tests the number of germinated spores per cm^2^ was seven times higher than those found in sterile tests. The percentage of germinated spores relative to the number of observed spores was also significantly higher when seawater microbes were present (**Figure 2B**). This indicates that bacterial presence notably improves *Ectocarpus* spore production and germination.

A morphological trait of *Ectocarpus* sp. (presence of upright filaments, the basis for the branched morphology) was also determined. The percentage of individuals with upright filaments after 3 weeks of growth under non-sterile or sterile conditions was calculated. In this case, 54% of the individuals grown under non-sterile conditions had already developed upright filaments whereas none individuals had developed these structures in sterile conditions (data not shown). This observation stresses the importance of bacteria for proper *Ectocarpus* development.

### Effect of Bacterial Isolates on *Ectocarpus* sp. Morphology and Reproduction

**FIGURE 3:**
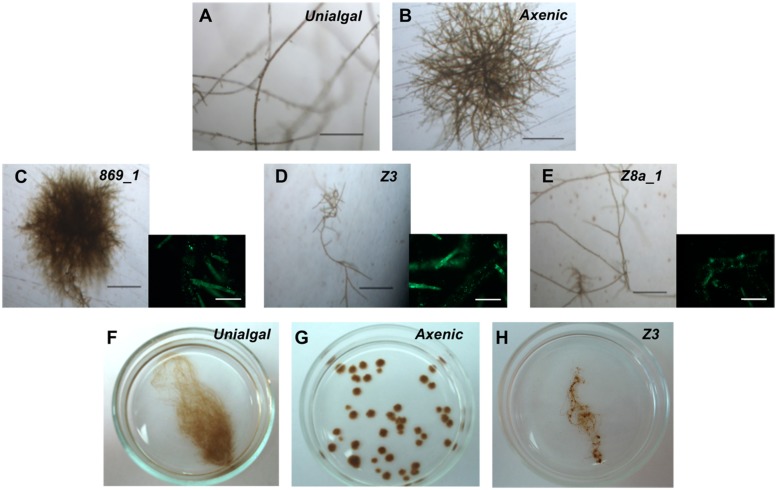
**Bacterial isolates effect on axenic *Ectocarpus* sp. upright filaments development.** **(A)** Portion of a representative *Ectocarpus* sp. individual showing filaments developed in a unialgal culture. **(B)** Axenic *Ectocarpus* sp. representative individual showing prostrate body development without any upright filaments. **(C)** *Ectocarpus* sp. representative individual grown in the presence of bacterial isolate 869_1 (*Kocuria rosea*) showing same morphology of axenic culture. **(D,E)** *Ectocarpus* sp. representative individuals grown in the presence of bacterial isolates Z3 (*Halomonas* sp.) and Z8a_1 (*Marinobacter* sp.), showing same phenotype of individuals in unialgal cultures with upright filaments developed. Images were taken 21 days post germination. All bars, 250 μm. **(C–E)** Accompanied by an epifluorescence microscopy image showing bacterial presence for each treatment. Bars, 50 μm. **(F–H)** Representative images of unialgal **(F)**, axenic **(G)** and bacterial inoculated (**H**, e.g., Z3) individuals after 6-week cultivation. While bacterial inoculated *Ectocarpus* recovered most of the typical branched morphology, axenic individuals grew as “small balls” showing no filaments.

Bacteria have a remarkable influence on the algal morphogenesis. After 21 days, upright filaments became visible in unialgal cultures (**Figure 3A**) but not in axenic *Ectocarpus* sp. (**Figure 3B**). In order to check for the presence of bacteria, we obtained DNA from unialgal culture supernatants and amplify 16S rRNA gene sequences by PCR. Then, we analyzed the digestion profile of the PCR amplicons. The presence of several electrophoretic bands demonstrated the presence of bacterial species in these *Ectocarpus* culture samples (**Supplementary Figure S1B**, right), and prompted us to isolate some of them. Nine different bacterial isolates were obtained from the surfaces of two different *Ectocarpus* unialgal strains, i.e., Ec 32 and Ec 524, and each of them was screened for its effects on *Ectocarpus* morphology. Although these cultivable bacteria do not reflect the entire *Ectocarpus* microbiota, they represent bacteria that are indeed associated to the alga. Thus, testing these isolates allowed description of the effects of at least part of the algal associated bacteria, but it should kept in mind that there might be a lot of missing bacteria that are uncultivable under the conditions used.

Seven of the nine isolates belonged to the *Proteobacteria* phylum while the other two were cataloged as *Actinobacteria* according to their 16S rRNA gene sequences (**Table 1**). Six out of these bacterial isolates triggered the development of upright filaments, being all members of the *Proteobacteria* phylum. Isolate 869_1 has no effect on such morphological trait (**Figure 3C**), whereas isolates Z3 and Z8a_1 were examples of the six isolates producing upright filaments (**Figures 3D,E**). After 6-weeks cultivation, bacterial inoculated *Ectocarpus* (**Figure 3H**) resembled the branched morphology of unialgal cultures (**Figure 3F**). On the other hand, the lack of upright filaments on axenic individuals gave them a “small ball”-like appearance (**Figure 3G**), which was far different to the other morphologies observed in conditions where bacteria were present. These findings confirm the initial observations that pointed out to the necessity of bacterial presence for *Ectocarpus* to develop its upright filaments and also show that a single bacterium can be enough to achieve this goal.

**Table 1:** Effect of nine bacterial isolates and their supernatants on morphology and reproduction of *Ectocarpus* sp. after 6 weeks of co-cultivation.

Isolate ID	Closest matching strain in NCBI database	% Sequence similarity	Filament inducing activity	Supernatant effect on morphology	Number of individuals/cm^2∗^
Z8a_1	*Marinobacter adhaerens* HP15 γ-Proteobacteria	99	Yes	No	46.3 ± 8
Z7	*Roseobacter* sp. 14III/A01/004 α-Proteobacteria	100	Yes	No	43.6 ± 5.3
Z3	*Halomonas* sp. Pper-Hx-1972 γ-Proteobacteria	100	Yes	Yes	74.3 ± 12.3
R8	*Marinobacter* sp. LCM-11γ-Proteobacteria	100	Yes	No	25.6 ± 5
R6a	*Antarctobacter* sp. LCM10-3α-Proteobacteria	99	Yes	No	35 ± 5.7
869_1	*Kocuria rosea* strain T1-2 Actinobacteria	100	No	No	11.3 ± 2
869_2	*Agrococcus citreus* strain IAM 15145 Actinobacteria	99	No	No	7.1 ± 3.2
Z1	*Alteromonas genovensis* γ-Proteobacteria	99	No	No	6.3 ± 2
R1	*Methylophaga* sp. γ-Proteobacteria	99	Yes	No	40 ± 7.6


*The number of individuals per cm^2^ is given with the standard deviation (±). ^∗^For axenic cultures 5.3 ± 1.5 and for unialgal cultures 39 ± 5.2.*

**FIGURE 4:**
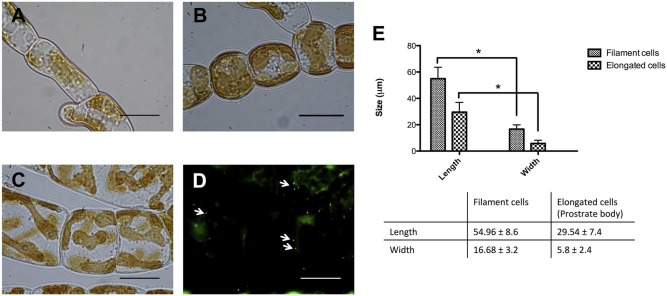
**Effect of bacterial isolates on cells types of *Ectocarpus* sp.** **(A)** Elongated cells (E cells) and **(B**) round cells (R cells) present in axenic and non-axenic individuals, respectively, as part of the prostrate body of the alga. **(C)** Typical upright filament cells in non-axenic individuals, in this case inoculated with isolate Z3**. (D**) Representative epifluorescence microscopy image of *Ectocarpus* individual inoculated with strain Z3. Arrows indicate bacterial presence on *Ectocarpus* sp. filaments determined by SYBR Green II staining. All bars correspond to 10 μm. **(E**) Comparison between length and width of elongated cells and filament cells. Cell lengths and widths of 60 individuals were measured. The ^∗^ indicates means statistically different at *p* < 0.05.

Bacterial effects directly influenced the cell types present in *Ectocarpus* sp. individuals. Under sterile conditions, *Ectocarpus* was composed of just two types of cells, elongated (E; **Figure 4A**) and round (R; **Figure 4B**). *Ectocarpus* in the presence of bacteria, in addition to contain R and E cells, displayed other types of cells that compose the upright filaments (**Figure 4C**). The cells in these filaments were very different from the R and E cells since they were larger, with an average of 55 and 17 μm of length and width, respectively; while E cells had an average of 30 and 6 μm for the same dimensions (**Figures 4A,C,E**). The effect of the presence of bacteria (in this case isolate Z3) in the formation of upright filaments was corroborated by epifluorescence microscopy (**Figure 4D**). Thus, by affecting *Ectocarpus* morphology, bacteria are also involved in cell differentiation processes crucial for algal development.

**FIGURE 5:**
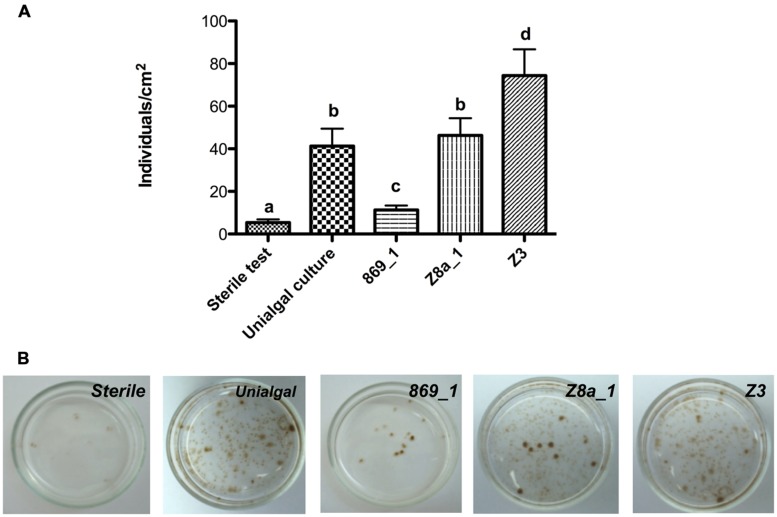
**Effect of bacterial isolates on *Ectocarpus* sp. reproduction.** **(A)** Number of individuals produced by *Ectocarpus* sp. after 6 weeks cultivation under axenic, unialgal conditions, or in the presence of different bacterial isolates. Data correspond to means ± SD (*n* = 12). Letters indicate means statistically different at *p* < 0.05 between all conditions. **(B)** Representative images showing the effects of bacteria on *Ectocarpus* sp. reproduction.

The effect of the presence or absence of bacterial isolates on *Ectocarpus* sp. reproduction was also addressed. In axenic algal cultures the number of individuals produced was around five per square centimeter versus the 25 to 70 counted when filament-producing bacteria (either in unialgal cultures, or as individual isolates) were present (**Figure 5**, third and last column in **Table 1**). While some bacterial isolates, -e.g., Z8a_1, Z7, and R1-, generated similar levels of individuals produced in unialgal cultures, Z3 increased significantly the number of individuals produced (**Figure 5** and **Table 1**). A clear correlation was found for the isolates that were capable to recover upright filaments development and their ability to trigger production of new individuals (**Table 1**). These results emphasize that, besides affecting morphology, bacteria are also relevant for *Ectocarpus* production of new germlings.

### Effects of Bacterial and Bacterial-Algal Co-cultures Supernatants on *Ectocarpus* sp. Morphology and Reproduction

**FIGURE 6:**
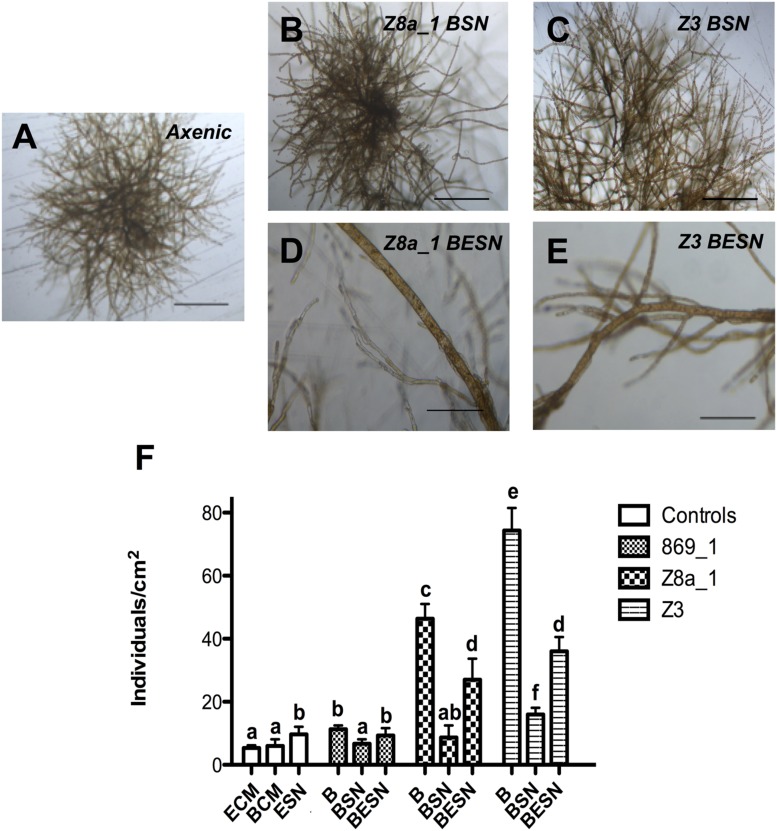
**Bacteria, and bacterial-algal co-culture supernatant effects on *Ectocarpus* sp. upright filaments development and reproduction.** **(A)** Absence of upright filaments development in axenic *Ectocarpus* sp. individuals grown without supernatant (SN) addition **(B,C)** Absence of upright filaments after addition of culture SN from bacterial isolates Z8a_1 (*Marinobacter* sp.) and Z3 (*Halomonas* sp.). **(D,E)** Presence of upright filaments after addition of co-culture SN from bacterial isolates Z8a_1 and Z3 plus *Ectocarpus* sp. All images recorded after 21 days of germination. Bars in **(A–C)** and **(D,E)** correspond to 250 and 100 μm, respectively. **(F)** Individuals per square centimeter produced by axenic *Ectocarpus* sp. grown in the presence of bacterial isolates **(B)**, supernatant of bacterial isolates (BSN) or supernatant from co-cultures of bacterial isolates and *Ectocarpus* sp. (BESN), after 6 weeks of cultivation. White bars represent control treatments with *Ectocarpus* culture medium (ECM), bacterial culture medium (BCM) and 1-week old supernatant from axenic *Ectocarpus* culture (ESN). All controls resembled axenic *Ectocarpus* morphology. Data correspond to means ± SD (*n* = 12). All treatments were compared between them. Letters indicate means statistically different at *p* < 0.05.

Growth culture supernatants from the nine isolated bacteria were obtained and tested for their ability to induce filaments development. None of the bacterial supernatants was capable of inducing growth of filaments (**Table 1**, **Figures 6B,C**), producing an algal morphology as that found for axenic cultures (**Figure 6A**), although bacterial supernatant from isolate Z3 modified *Ectocarpus* early development (**Supplementary Figure S4**). The effect of supernatants obtained from co-cultures of bacterial isolates and *Ectocarpus* sp. were then tested. Some of these co-culture supernatants did recover upright filament development, but this effect was only achieved for those co-cultures of bacterial isolates that were able to induce filaments presence (**Figures 6D,E**).

Concerning reproduction of *Ectocarpus* individuals, bacterial supernatants did not increase the number of individuals, except for isolate Z3 supernatant which slightly increased the individuals produced with respect to the control (**Figure 6F**). Co-culture supernatants had the same effect of the isolates from which these supernatants were produced, but with a lower impact compared to direct exposure to bacteria (**Figure 6F**). According to these results, bacterial effects on *Ectocarpus* morphology and reproduction are accomplished by active interaction with the alga, needing both organisms to be in the same culture. Furthermore, the compound (s) responsible of algal upright filaments emergence and stimulation of reproduction is (are) produced during bacterium-alga co-cultures and is (are) released to the media.

### Effect of Bacteria on *Ectocarpus* sp. Released Metabolites, i.e., Exometabolome

**FIGURE 7:**
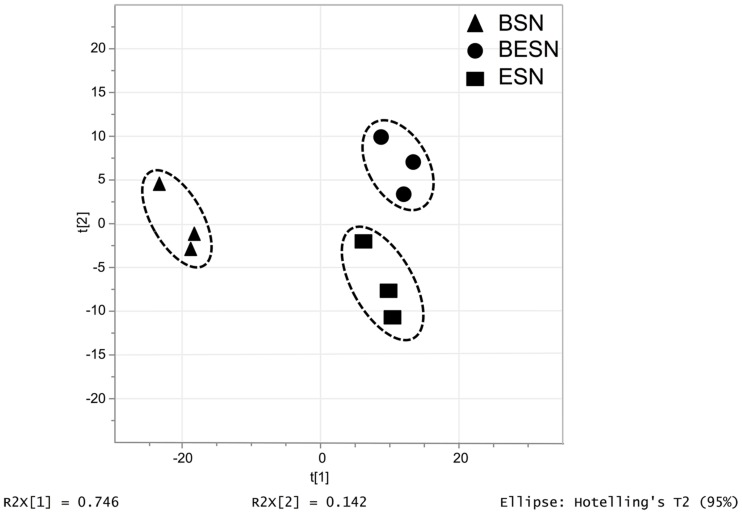
**Principal Component Analysis (PCA) plot carried out for metabolite profiles (exometabolome) detected from supernatants from the bacterial isolate *Halomonas* sp.** Z3 culture (BSN), strain Z3 and *Ectocarpus* sp. co-culture and axenic *Ectocarpus* sp. culture (ESN). The score plot was obtained using 320 monoisotopic peaks quantified by UPLC-MS in positive ionization mode. All metabolites were considered for PCA (*p*-value < 0.05) generated by SIMCA-P v12.0.

In order to get some insight about the bacterial effect on the metabolism of the alga, the metabolite profiles of exudates from axenic *Ectocarpus* sp. alone, a bacterial isolate alone, and the combination of both organisms were determined by ultra-high pressure liquid chromatography (UPLC) coupled to mass spectrometry (MS). In this case, bacterial isolate Z3 was chosen to perform the evaluation because it had filament-inducing activity and it was the one with the greatest effect on reproduction (**Table 1**). The samples were taken after 3 weeks of co-culturing since at this time point filaments were already developed. A global metabolite profiling by LC-MS-MS in positive ion mode provided the more informative set of data, with 320 signals, and was used for further analysis. Multivariate analysis of these exometabolome profiles revealed specific clustering for the three conditions analyzed, with a clear separation of a three distinct groups along the two axis, explaining 76.4 and 14.2% of the variance as it is shown by PCA plot (**Figure 7**). These data indicate that *Ectocarpus* sp. plus this bacterium released a set of metabolites that is distinct from those generated by the same bacterium and alga growing alone.

## Discussion

Algae provide an advantageous environment for proliferation of bacteria, some of which have already been shown to have positive effects on their hosts (reviewed in Singh and Reddy, 2014). For brown algae, putative beneficial effects of bacteria on development still remain to be experimentally tested and fully established. Regarding to *Ectocarpus*, microbiota relevance under abiotic stress was recently investigated (Dittami et al., 2015). In this context, the present study goes deeper on previous observations about the importance of bacteria for these marine organisms. This work demonstrates that bacteria influence morphology and reproduction of the brown algal model *Ectocarpus* sp. Typical branched morphology of this alga is clearly dependent on the presence of bacteria. This finding is consistent with previous studies on green algae, as members of *Ulvaceae* lose their typical morphology when cultured under axenic conditions (Provasoli and Pintner, 1980) but recovered it when inoculated with appropriate morphogenesis-inducing bacterial isolates (Matsuo et al., 2003; Marshall et al., 2006). For red algae, the role of bacteria on morphological development had been also demonstrated (Singh et al., 2011; Fukui et al., 2014). The fact that the three major groups of multicellular algae are influenced in their morphology by bacteria, strongly suggest that this type of interaction has been relevant for these organisms during their evolution. The effect of microorganism communities contained in natural seawater on axenic *Ectocarpus* sp. (**Figure 1**) resembled the effect of isolated bacteria. This is significant because it validates the use of single bacterial isolates as a proxy of what the alga could found in the field.

In the present study, we evaluated the effect of nine bacterial isolates obtained from unialgal laboratory cultures of *Ectocarpus* sp. This rather low number of bacterial isolates may be explained by the constraints imposed to this alga under laboratory conditions. The *Ectocarpus* strains used to isolate bacteria has been kept under laboratory conditions for long time (years) and they have been exposed to conditions (including antibiotic treatments), which decreased bacterial diversity and abundance at an extent difficult to determine.

It might appear that there could be some specificity in the ability of these bacterial isolates to have an effect on *Ectocarpus* morphology because only proteobacterial isolates showed effects on this alga. Although *Proteobacteria* has been shown as a dominant phylum in other studies describing bacterial communities associated with algae (Hengst et al., 2010; Burke et al., 2011a; Hollants et al., 2013) we cannot discard a possible bias in the bacterial isolation procedure, which led to preferential selection of these microorganisms. In order to clarify this issue, we did a gross survey on bacterial diversity associated to field and laboratory *Ectocarpus*. The majority (72 and 56% for field and laboratory samples, respectively) of the sequences analyzed were affiliated to *Proteobacteria* (**Supplementary Figure S5A**), which is consistent with the dominance of this phylum between the bacterial isolates reported here. Remarkably, the recent study of Dittami et al. (2015) also reports the dominance of *Proteobacteria* associated to laboratory strains of *Ectocarpus.* The similarity in abundances at phylum level between field and laboratory samples supports the idea that what we observed in laboratory specimens could be applied to the field. Interestingly, most of the bacterial strains isolated in this work were detected using this culture-independent approach in both field and laboratory algae (**Supplementary Figure S5B**). Again, the abundances of these bacteria in field and laboratory *Ectocarpus* were quite similar. In general, the bacterial isolates correspond to 11% of total microbiota. Most isolates are low-abundance bacteria (less than 1%) except for the *Roseobacter* representative, which is very abundant when consider all samples together (**Supplementary Figure S5B**), although its abundance is rather low in several samples (**Supplementary Figure S5C**).

It should be kept in mind that this report evaluated the role of bacteria using a culture-dependent approach. There are studies that have established that only a small proportion of bacteria can be cultivated using conventional methodologies (Whitman et al., 1998; Fry, 2000; Handelsman, 2004). In this context, the results showed in this work might apply to a small part of bacteria thriving on the surface of *Ectocarpus*, although they reflect bacteria indeed associated with this alga.

A deeper exploration of the taxonomic affiliation of bacterial isolates capable to induce filaments development showed that, apart of belonging to the *Proteobacteria* phylum, there is no further taxon specificity in the effects observed. Bacterial isolates producing morphology/reproduction effects are distributed among several families and genera. This observation has been also reported for green algae (Nakanishi et al., 1996; Marshall et al., 2006). These studies reported that several bacterial genera are capable to influence morphology of *Ulva pertusa* and *U. linza*, including genus *Vibrio, Pseudomonas, Halomonas, Escherichia* and some Gram-positive bacterial genera. In our study, we also found a *Halomonas* isolate (strain Z3) having a strong impact on morphology. In contrast, isolates belonging to the genera *Antarctobacter* (R6a), *Marinobacter* (Z8a_1, R8), and *Methylophaga* (R1), are for the first time described to influence macroalgal development. On the other hand, the two *Actinobacteria* isolates studied here did not have any effect on *Ectocarpus* development, although the impact of member of this phylum on green algal morphology has been reported (Nakanishi et al., 1999; Marshall et al., 2006).

*Ectocarpus* sp. early sporophyte development has been already described. Le Bail et al. (2008b) reported that sporophytes grow as prostrate filaments composed of two cell types, E and R. These cells form the prostrate body of the alga. If the growth conditions are favorable, upright filaments emerge after a few days, contributing to the establishment of an overall filamentous architecture (Ravanko, 1970). In the present study, the upright filaments appearance was found to be a bacterial modulated process. When bacteria were not present in the culture medium, *Ectocarpus* sp. developed its prostrate body without any upright filaments (**Figure 3**) producing only E and R cells (**Figures 4A–E**). In contrast, when axenic *Ectocarpus* sp. was cultivated in culture medium containing microorganisms, or with bacterial isolates (**Figure 3**), it developed upright filaments and recovered most of its filamentous morphology (**Figure 5**). Although the influence of bacteria on algal morphology had been reported, it is relevant to stress that the effect of bacteria on the appearance is not only on the filaments *per se*, but also in the new cell types required to form these structures. The cells composing the filaments are very different from those of the prostrate body (**Figure 4**), which means that bacteria are capable of triggering cell differentiation mechanisms in the alga. In this regard, plant hormones represent very good candidates to produce these kinds of effects. These compounds control plant growth by affecting the spatial and temporal expression of genes involved in cell division, elongation, and differentiation. Pedersen (1968), early suggested that *E. fasciculatus*, a sister species of *Ectocarpus* sp. needs cytokinins in order to grow normally under culture conditions. In *Ectocarpus* sp. it had been suggested that auxins could be involved on upright filaments appearance by repressing its emergence (Le Bail et al., 2010). Although phytohormones presence on macroalgae have been reported (Stirk et al., 2003), to date there is no evidence of bacterial phytohormones production having a direct effect on algal development, despite it is already known that marine bacteria can produce these compounds (Maruyama et al., 1986, 1990).

A possible explanation to the results obtained with the supernatant essays is that all the bacterial isolates capable of inducing filament appearance secrete filament-inducing factor(s) (e.g., phytohormones) into the culture supernatant only when *Ectocarpus* sp. is also present (co-cultures). When bacterial isolates were grown alone their supernatants did not have an effect in morphology or reproduction (**Figure 6**). In this context, it has been proposed that *Ectocarpus* could manage to produce phytohormones in association with bacteria (Dittami et al., 2014), and the same has been predicted recently for diatoms and their interaction with bacteria (Amin et al., 2015). The production and exchange of chemicals cues between algae and bacteria seems to be critical for the wellbeing of these organisms in natural conditions. Nevertheless, in other studies bacterial supernatants have been shown to be sufficient in modulating algal development. In the green alga *Monostroma oxyspermun*, supernatants of bacterial cultures recover the normal morphology of the alga (Matsuo et al., 2003). Matsuo et al. (2005) identified this exogenous growth factor as thallusin, produced by bacteria belonging to *Bacteroidetes* phylum. Because of their evolutionary distance, it is not surprising that the mechanisms involved in the effect of bacteria on green and brown algae could be different.

It is not clear if contact between bacteria and *Ectocarpus* sp. is required for morphology and reproduction to be affected. The reported observations do not rule out the possibility that bacteria need not to be in contact with the alga but just closely enough to communicate with each other and produce the compound(s) responsible for the described effects. On this regard, some PGPB have been shown to exert their effect by production of volatile organic compounds (VOCs) without requiring direct contact with the plant (Gutiérrez-Luna et al., 2010; Meldau et al., 2013). These evidences suggest that a similar mechanism could be involved in the described effects of bacteria on *Ectocarpus* development. What is clear is that the presence of both organisms in the same culture is needed in order to produce filaments development, which implies that some interaction exists between bacteria and alga. The effect accomplished by the co-culture supernatant in morphology and reproduction means that the compound(s) responsible for this phenomenon is (are) secreted and stable in the culture medium, at least for some time. When comparing algal individuals produced by axenic *Ectocarpus* sp. exposed to direct bacterium inoculation versus the exposure to co-culture supernatants, direct inoculations have an stronger impact than co-culture supernatants. This suggests that the compound(s) responsible for the effects was (were) not stable for a long time in the culture medium, so the permanent presence of bacterium (and concomitant continuous production) seems to be required to produce more pronounced effects.

The influence of bacteria on *Ectocarpus* physiology was reflected by the results of the metabolomic approach shown in this work. The recorded data demonstrated that *Ectocarpus* sp. associated with a single bacterium produces a different metabolite profile compared to those of axenic alga. However, which compounds cause the effects described was not assessed. Other reports combined metabolomics with bioassays thus narrowing down the metabolome to one biologically active compound (Matsuo et al., 2005; Schroeder et al., 2006). The untargeted analysis performed here does not yield functional information, unless it is combined with a bioassay as well. For the majority of metabolites that were detected, both the identity and the function in the *Ectocarpus*-bacterium interaction, is largely unknown. However, it is quite clear that the impact of bacteria on *Ectocarpus* metabolomic profile shows that bacterial influence is exerted at several levels of algal physiology.

In summary, this article supports the importance of bacteria for reproduction, growth and development of the brown algal model *Ectocarpus* sp. The range of bacteria that affect development on *Ectocarpus* sp. could confer ecological flexibility to the alga. This may be important since this alga inhabits worldwide along temperate coastlines, where it can grow on either rocky and/or artificial substrates or epiphytically on other algae thus being challenged by very different bacterial communities. The mechanisms involved in this interaction are presently unknown, but at least some communication is required to display the effects described. Bacterial impacts on physiology were also highlighted since one bacterial isolate could drive major changes in the algal exometabolomic profile. Altogether, the data reported in this study along with the molecular tools already available for *Ectocarpus* sp. open a new window in the study of algal host–microbes interactions.

## Author Contribution

JT performing experiments, conducting the work, design of the work, analysis, interpretation of data for the work, responsible for the integrity of the work as a whole, final approval of the version to be published. BG design of the work, interpretation of data for the work, critically revising the final approval of the version to be published. SG performing experiments, analysis, interpretation of data for the work. PP design of the work, analysis, interpretation of data for the work, ensuring that questions related to the accuracy or integrity of any part of the work are appropriately investigated and resolved, critically revising the final approval of the version to be published, responsible for the integrity of the work as a whole. JC design of the work, analysis, interpretation of data for the work, ensuring that questions related to the accuracy or integrity of any part of the work are appropriately investigated and resolved, critically revising the final approval of the version to be published, responsible for the integrity of the work as a whole.

## Conflict of Interest Statement

The authors declare that the research was conducted in the absence of any commercial or financial relationships that could be construed as a potential conflict of interest.

## Footnotes

1 http://blast.ncbi.nlm.nih.gov/Blast.cgi

2 http://rsb.info.nih.gov/ij/

3 http://camera.calit2.net/
