# Supplementary material for: Microbiota Influences Morphology and Reproduction of the Brown Alga Ectocarpus sp
Source: Front Microbiol. 2016 Feb 24;7:197. doi: 10.3389/fmicb.2016.00197 (PMC4765120; doi:10.3389/fmicb.2016.00197)
Supplement: Supplementary file 1 [file Image_1.PDF]

Supplementary Material

Microbiota influences morphology and reproduction of the brown alga *Ectocarpus* sp.

Tapia, J.E., González, B., Goulitquer, S., Potin, P., Correa, J.A.*

*** Correspondence:**

Dr. Juan A. Correa
Pontificia Universidad Católica de Chile
Departamento de Ecología
Laboratorio de Algas
Alameda 340
Santiago, 8331150, Chile
jcorrea@bio.puc.cl

Supplementary Figures

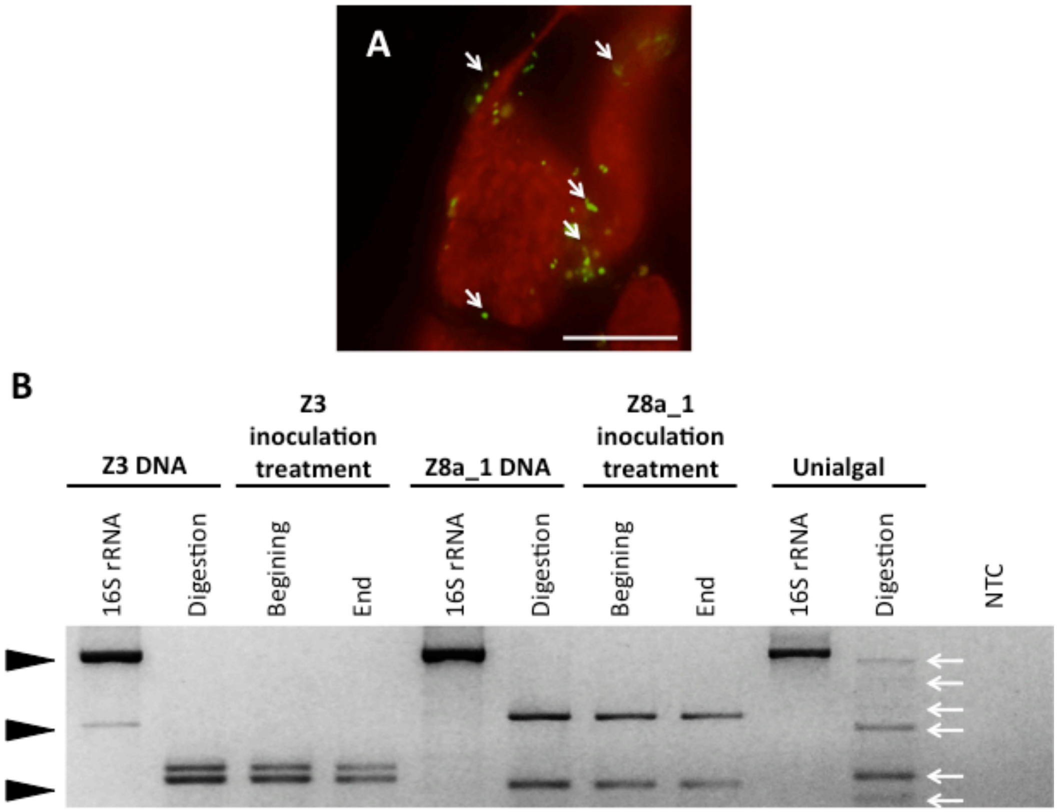

Supplementary Figure S1. Detection of bacteria in unialgal cultures. **A.** SYBR green II stained *Ectocarpus* cells showing the presence of bacteria at the end of inoculated experimental treatments with the bacterial isolate Z8a_1. **B.** 16S rRNA gene DNA and their *Alu*III digestion PCR products. *Alu*III digestion procedure was performed according to manufacturer's protocol (Invitrogen). PCR were performed with the indicated DNA templates and the primers 8F and 1492R. Arrows indicate multiple bands in the 16S rRNA gene amplicon digestion of DNA from the unialgal culture supernatant, indicative of the presence of several bacteria. Arrowheads indicate the position of 1500, 500 and 250 bp DNA size markers. NTC, non-template control.

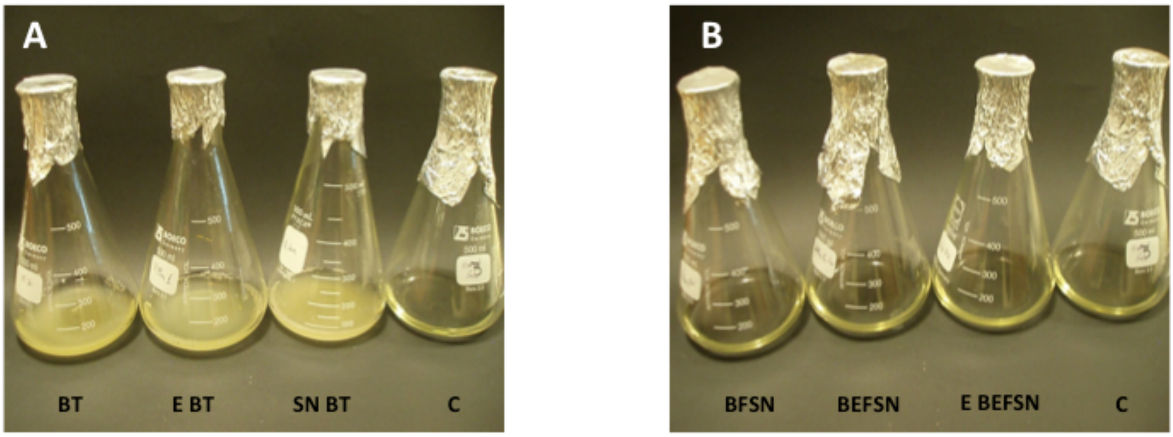

Supplementary Figure S2. Growth tests of inoculated and non-inoculated algal experiments. Growth tests were performed in Zobell growth medium inoculated with samples from inoculated (**A**) and non-inoculated (**B**) treatments. Looking at the turbidity of the media into the flasks we can observe the presence of bacteria in the inoculated treatments and the efficiency of the filtration and pasteurization processes in the non-inoculated treatments. BT (direct bacterial inoculum), E BT (inoculated *Ectocarpus*, end of treatment), SN BT (supernatant from inoculated *Ectocarpus*, end of treatment), C (control, non inoculated axenic *Ectocarpus* supernatant), BEFSN (filtered supernatant from inoculated *Ectocarpus* (co-culture), E BEFSN (*Ectocarpus* exposed to filtered supernatant from inoculated *Ectocarpus*), C (control).

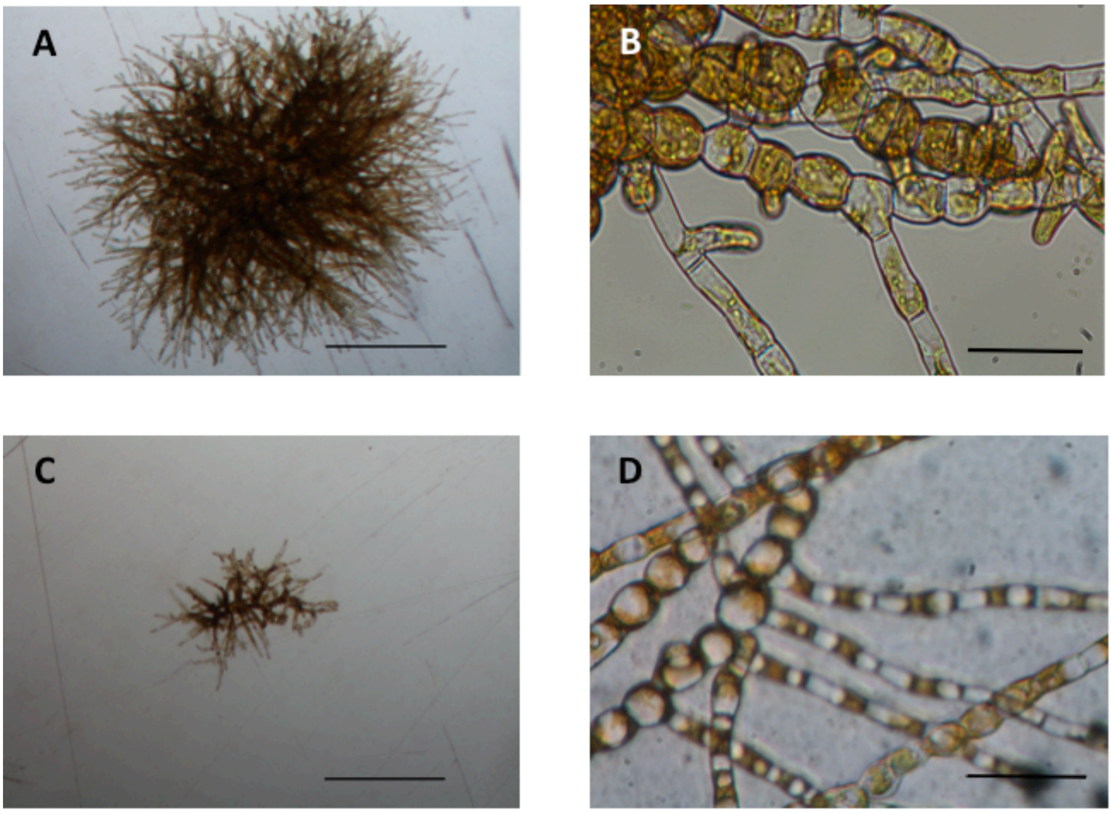

Supplementary Figure S3. Comparison between two culture conditions in the growth of axenic *Ectocarpus* sp. **A**. Axenic *Ectocarpus* sp. representative individual showing prostrate body development without any upright filaments growing in one-week old alga-bacteria co-culture supernatant. **B**. Cells from axenic *Ectocarpus* in one-week old alga-bacteria co-culture supernatant showing typical pigmentation and chloroplast distribution. **C**. Axenic *Ectocarpus* sp. representative individual showing prostrate body development without any upright filaments growing under starvation stress culture condition. The culture medium used in this case was not supplemented with nutrients (nitrate and phosphate). The alga in this condition grew significantly less compared with the control. **D**. Cells from axenic *Ectocarpus* under stress conditions show less pigmentation and shrinkage of chloroplasts. Images were taken 21 days after germination. Bars in A and C, 500 µm. Bars in B and D, 20 µm.

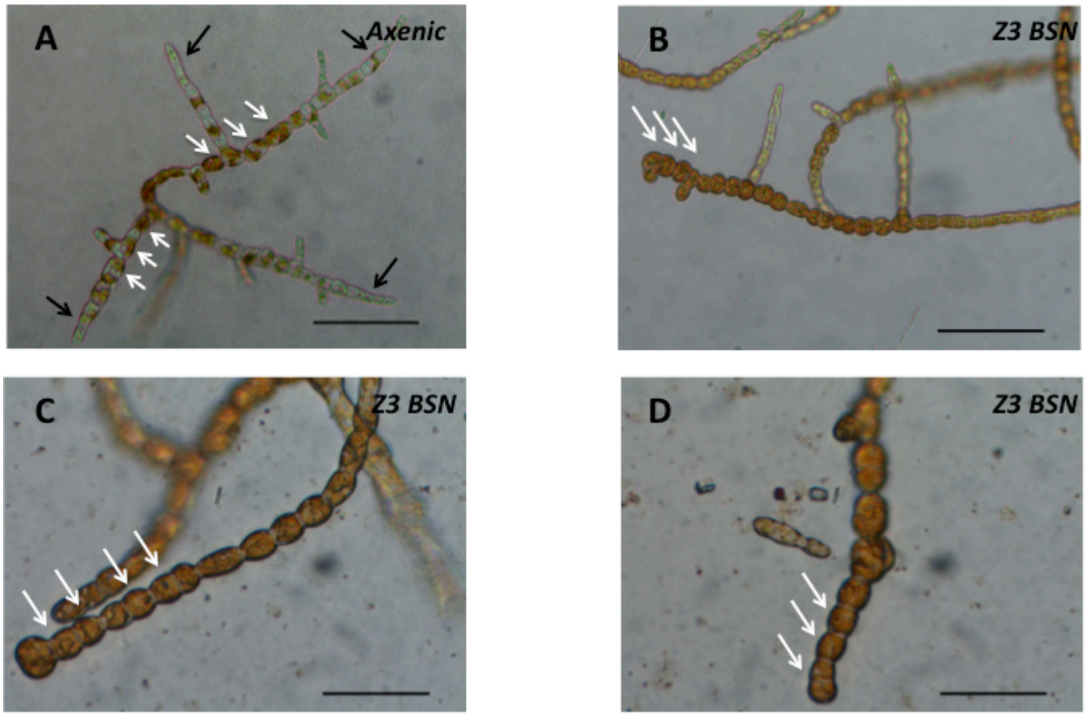

Supplementary Figure S4. Effect of isolate Z3 supernatant (SN) on the early development of *Ectocarpus* sp. **A.** Axenic *Ectocarpus* (7 days after germination) showing regular development with round (white arrows) and elongated (black arrows) cells, where round are concentrated on the center of the structure and elongated ones are at the extremes. **B-D.** Z3 supernatant alters early development of *Ectocarpus.* Individuals grow with more round cells even at the extremities of the prostrate body (white arrows). Bars in A-B and in C-D correspond to 50 and 25 µm, respectively.

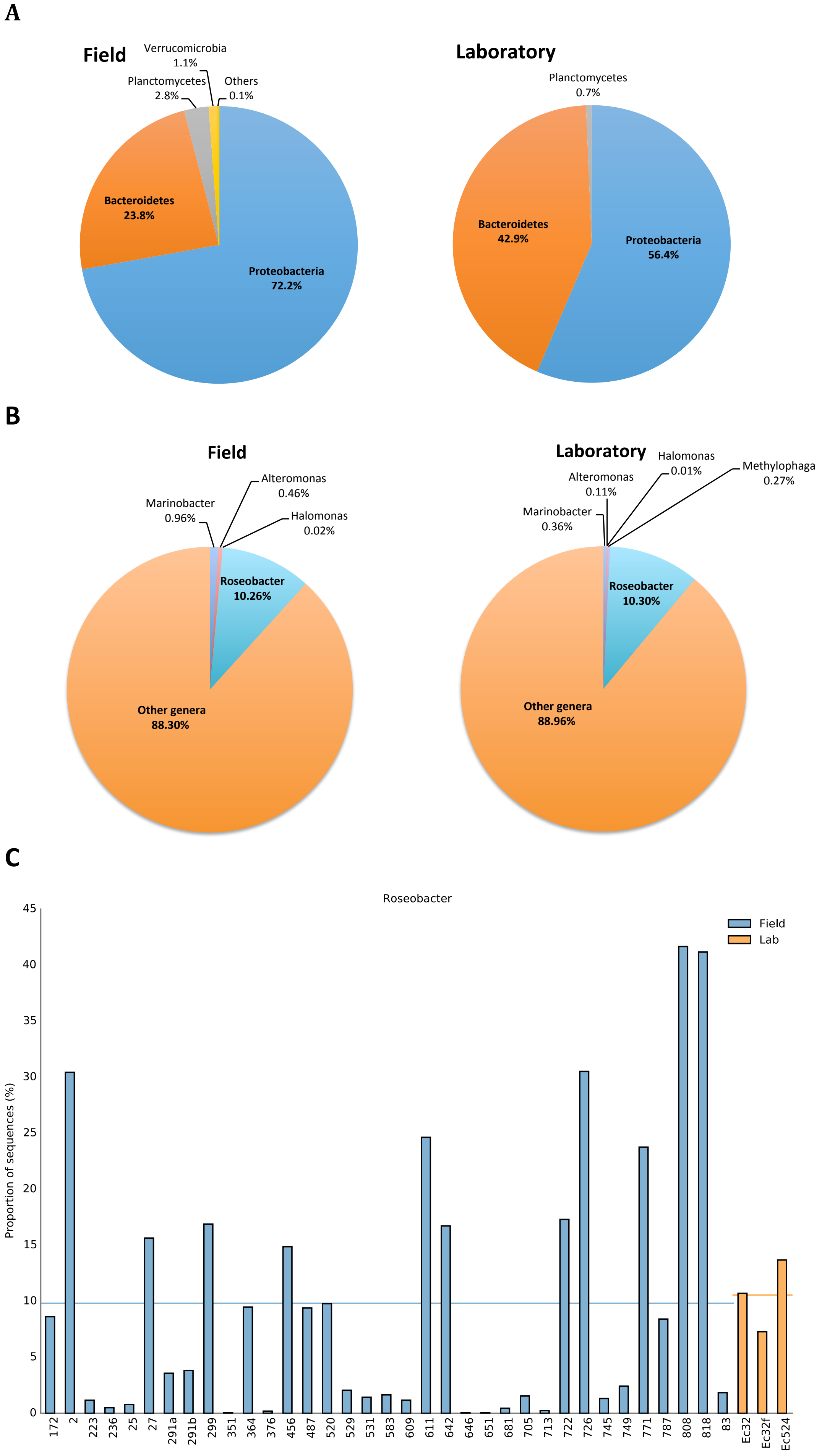
A
Field
Verrucomicrobia 1.1%
Planctomycetes 2.8%
Others 0.1%
Bacteroidetes 23.8%
Proteobacteria 72.2%
Laboratory
Planctomycetes 0.7%
Bacteroidetes 42.9%
Proteobacteria 56.4%
B
Field
Alteromonas 0.46%
Marinobacter 0.96%
Halomonas 0.02%
Roseobacter 10.26%
Other genera 88.30%
Laboratory
Halomonas 0.01%
Methylophaga 0.27%
Alteromonas 0.11%
Marinobacter 0.36%
Roseobacter 10.30%
Other genera 88.96%
C
Roseobacter
Field
Lab
Proportion of sequences (%)
0
5
10
15
20
25
30
35
40
45
172
2
223
236
25
27
291a
291b
299
351
364
376
456
487
520
529
531
583
609
611
642
646
651
681
705
713
722
726
745
749
771
787
808
818
83
Ec32
Ec32f
Ec524

Supplementary Figure S5. Prevalence of the bacterial isolates obtained in this work in field and laboratory samples of *Ectocarpus*. Thirty five samples from different places of the Chilean coast were obtained plus 3 unialgal laboratory samples (Ec32 and Ec 524 correspond to strains which are maintained in P. Universidad Católica facilities plus Ec32f, which is the same strain Ec32 but maintained in the Station Biologique de Roscoff, under different conditions). For all algal samples, bacterial DNA was extracted according to Burke *et al.* (2009). Bacterial 16S V3-V4 rRNA gene sequences were amplified and sequenced on the Illumina (San Diego, CA, USA) MiSeq platform and processed to obtained taxonomic affiliations as described in Dittami *et al.* (2015). After data processing, 7.1 million bacterial 16S rRNA gene reads with an average of 187292 ± 70944 (s.d.) sequences per sample were obtained. Overall, 769 operational taxonomic units (OTU) were found at 99% identity threshold **A.** Distribution of phyla present in field and laboratory samples. **B.** Distribution of bacterial isolates in laboratory and field samples. Sequences representing *Antarctobacter*, *Kocuria* and *Agrococcus* genera were not found. **C**. Specific *Roseobacter* abundances in all samples analyzed. Blue and orange lines indicate mean abundance for field and laboratory samples, respectively.

Supplementary Table

Supplementary Table S1. GenBank accession numbers of 16S rRNA gene sequences from bacterial isolates obtained in this work.

Isolate ID	*GenBank accession number*
Z8a_1	KT461661
Z7	KT461667
Z3	KT461664
R8	KT461666
R6a	KT461665
869_1	KT461662
869_2	KT461663
Z1	KT461668
R1	KT461669

Supplementary Material References

Burke C, Kjelleberg S, Thomas T (2009) Selective extraction of bacterial DNA from the surfaces of macroalgae. *Appl Environ Microbiol* **75**: 252-256

Dittami SM, Duboscq-Bidot L, Perennou M, Gobet A, Corre E, Boyen C, Tonon T (2015) Host-microbe interactions as a driver of acclimation to salinity gradients in brown algal cultures. *ISME J* doi:10.1038/ismej.2015.104
